# An estimate of extracellular vesicle secretion rates of human blood cells

**DOI:** 10.1002/jex2.46

**Published:** 2022-06-02

**Authors:** Martin Auber, Per Svenningsen

**Affiliations:** ^1^ Department of Molecular Medicine University of Southern Denmark Odense Denmark

**Keywords:** cell types, EV secretion rate, mitochondrial metabolism

## Abstract

Extracellular vesicles (EVs) have been implicated in the intercellular transfer of RNA and proteins through cellular secretion into the extracellular space. In blood plasma, circulating EVs are mainly derived from blood cells; however, factors that control plasma EV abundance are largely unknown. Here, we estimate the EV secretion rates for blood cell types using reported values for cell‐specific plasma EV abundances and their parental cell's ubiquity in healthy humans. While we found that plasma contains on average ∼2 plasma EVs/cell, the cell‐specific EV‐to‐cell ratio spanned four orders of magnitude from 0.13 ± 0.1 erythrocyte‐derived EVs/erythrocyte to (1.9 ± 1.3) × 10^3^ monocyte‐derived EVs/monocyte. The steady‐state plasma EV level was maintained by an estimated plasma EV secretion rate of (1.5 ± 0.4) ×  10^12^ EVs/min. The cell‐specific secretion rate estimates were highest for monocytes (45 ± 21 EVs/cell/min) and lowest for erythrocytes ((3.2 ± 3.0) ×  10^−3^ EVs/cell/min). The estimated basal cell‐specific EV secretion rates were not significantly correlated to the cell's lifespan or size; however, we observed a highly significant correlation to cellular mitochondrial enzyme activities. Together, our analysis indicates that cell‐specific mitochondrial metabolism, for example, via reactive oxygen species, affects plasma EV abundance through increased secretion rates, and the results provide a resource for understanding EV function in human health and disease.

## 1 INTRODUCTION

The blood plasma concentration of the membrane‐enclosed extracellular vesicles (EVs) in healthy humans is ∼10^10^ EVs/ml (Johnsen et al., 2019), maintained by continuous turnover of cellular secreted EVs. In healthy persons, the plasma EVs are primarily derived from blood cells such as platelets, T‐cells, and B cells (Koliha et al., 2016; Li et al., 2020); however, alterations in the cellular environment, such as hypoxic conditions (Bister et al., 2020) and exercise (Brahmer et al., 2019) cause higher EV secretion rates from specific cells. The higher secretion rate can affect the plasma EV concentration and composition, providing easy access to biomarkers of diseases, for example, cancers (Witwer et al., 2013). Nonetheless, the factors determining the plasma EV abundance in healthy humans are not well‐established.

Plasma EV abundance is determined by their secretion and elimination rates, and most of our knowledge on this EV turnover in blood plasma is based on animal studies (Kang et al., 2021). In mice, intravascular‐administered EVs have a plasma half‐life of ∼30 min independent of the EV donor cell type (Kang et al., 2021). While the EV elimination rate is not significantly affected by the administered EV dose (Imai et al., 2015), macrophage depletion increases the circulatory time of injected EVs by ∼5‐fold (Imai et al., 2015). This suggests a high capacity to clear plasma EV from circulation. Consistent with this, macrophages can interiorize the equivalent of their cell surface area every 33 min (Steinman et al., 1976), suggesting that they are essential for the overall plasma EV concentration. In humans, the plasma EV half‐life has not yet been determined directly. The half‐life of platelet‐derived EVs was indirectly determined in severely thrombocytopenic patients receiving platelet transfusion to 5.5 h (Rank et al., 2011). The infused platelets are, however, likely to continue to produce EVs, thereby overestimating their half‐life (Rank et al., 2011). Corroborating this notion, the increased plasma EV concentration induced by acute interventions, such as dobutamine stress echocardiogram (Augustine et al., 2014) or exercise (Frühbeis et al., 2015), were normalized within 1 h in humans. The plasma EV elimination rate in humans is, thus, likely to be comparable to mice and independent of parental cell type, suggesting that plasma EV concentration and composition are critically dependent on cell‐specific EV secretion rates.

Cell‐specific EV secretion rates have not yet been determined *in vivo*. Still, cultured cells show high EV secretion rate variations, for example, the hepatocellular carcinoma cell line HepG2 secrete ∼0.08 EVs/cell/min – that is, ∼1 EV per 15 min (Son et al., 2015), while epithelial cell lines MCF7, MDA‐MB‐231, MCF10A secrete ∼1‐3 EVs/cell/min (Chiu et al., 2016). This high difference in cell type‐specific EV secretion rates suggests that the plasma EV concentration is not proportional to cell abundance but dependent on cell‐specific mechanisms that contribute to the overall basal EV secretion rate. To elucidate cellular mechanisms that affect plasma EV concentration, we integrated reported values for the circulating EV levels and their parental cells’ abundance and phenotype to create a quantitative estimate of secretion rates of the most abundant cell‐derived EVs in the human blood plasma.

## 2 METHODS

### 2.1 Plasma EV abundance, size, and mass

Lipoprotein particles outnumber plasma EVs by six to seven orders of particle concentration (Johnsen et al., 2019). We, therefore, used data from five studies (Brennan et al., 2020; Buschmann et al., 2018; Holcar et al., 2020; Jamaly et al., 2018; Mørk et al., 2017) that used ultracentrifugation or immune‐affinity techniques to deplete human plasma of lipoproteins to assess the mean plasma EV concentration and size by nanoparticle tracking analysis (NTA). We did not make a distinction between small EVs and large EVs. The data used for estimation of the plasma EV concentration are shown in File S1. The plasma and blood volumes for young, healthy males were derived from Sender et al. (Sender & Milo, 2021), references therein, and are shown in File S1.

### 2.2 Mean plasma EV residence time

As stated in the introduction, the data on the plasma EV half‐life in humans is consistent with animal studies, and we inferred the mean plasma residence time (MRT) for plasma EVs from mouse studies of administered EV (Imai et al., 2015; Matsumoto et al., 2017; Morishita et al., 2015; Takahashi et al., 2013). The plasma MRT was calculated to 41 ± 7 min (File S1). *Ex vivo* handling of EVs, for example, freeze‐thaw cycles, cause a higher degree of phosphatidylserine surface exposure (Ayers et al., 2011; Johnson et al., 2014) that may increase phagocytosis rate (Ayers et al., 2015), and the administered EVs may underestimate the MRT of endogenously produced EVs.

### 2.3 Cell types chosen for analysis

EVs are highly heterogeneous with respect to their protein and RNA cargo both between and within cell types, for example, cells overexpressing EV reporter proteins do not produce EVs that are uniformly labelled with the marker (Sung et al., 2020). Thus, the cellular origin of the plasma EVs based on a single cell marker may not capture all EVs derived from a single cell type. For example, platelet‐derived EVs were shown to be less (Fendl et al., 2016) or more (Palviainen et al., 2020) abundant than erythrocyte‐derived EVs by flow cytometry of EVs using anti‐CD41 or anti‐CD61 antibodies, respectively. Nonetheless, by multiplex bead‐based flow cytometry, the surface proteins on plasma EVs indicated that they are mainly derived from platelets, B cells, and T cells in plasma from healthy humans (Koliha et al., 2016; Wiklander et al., 2018). In addition to proteins, EVs contain functional RNAs such as mRNA (Valadi et al., 2007). Although the microRNAs abundance is probably low (Chevillet et al., 2014), other RNA species are more abundant, and it has been estimated that each EV contains ∼100 RNA molecules (Piffoux et al., 2021). By computational deconvolution approaches, the matching of several tissue/cell‐specific genes profiles with the RNA profile of isolated EVs has been used to determine the cellular source of EVs in plasma and urine (Li et al., 2018, 2020, 2021; Zhu et al., 2021). Like the flow cytometry data based on surface markers, the RNA‐based EV data indicate that most, that is, 99.8% of the circulating EVs, are blood cell‐derived (Li et al., 2020). To minimize potential interference from intercompartmental barriers, such as the blood‐brain barrier, we, therefore, focused on blood cells and their plasma EV abundances and used data from exoRBase 2.0 (see File S1) on the blood cell origin of plasma EVs from 118 healthy persons (Lai et al., 2021; Li et al., 2018). We grouped some cell types, for example, CD4 memory cells, to simplify the presentation.

### 2.4 Cellular abundance, lifespan, and turnover

Data for cell‐type abundance, mass, and lifespan were collected from a recent census of human cellular turnover (Sender & Milo, 2021) and are shown in File S1. We added additional data for Natural Killer (NK) cells and platelets. NK cell's lifespan was estimated using data from Zhang et al. (Zhang et al., 2007), and for the platelet's abundance and lifespan, we used data from Bianconi et al. (Bianconi et al., 2013), Wessels et al. (Wessels et al., 1985) and Harker et al. (Harker et al., 2000). The average and total platelet mass were calculated by multiplying platelet density (Martin et al., 1983) with average platelet volume (Harker et al., 2000) and total platelet number (Bianconi et al., 2013), respectively.

### 2.5 Mitochondrial phenotype

The immune cells’ mitochondrial phenotype data were collected from Rausser et al. (Rausser et al., 2021). The data include direct measurements of enzymatic activities and mitochondrial DNA copy numbers in B cells, CD4, CD8, monocytes, neutrophils, and NK cells from healthy humans (Rausser et al., 2021). We did not identify comparable data for platelets, and this cell type was excluded from the analysis. Moreover, erythrocytes have no or very few mitochondria (Zhang et al., 2011), and their mitochondrial phenotype was therefore set as zero.

### 2.6 Statistic

Data are mainly presented as mean ± standard deviation. In cases of large variations, data are presented as multiplication factors. The uncertainty estimates were derived from reported values of the individual measurements, and the overall uncertainty was calculated based on error propagation of all individual components using the uncertainties package (Lebigot, 2021) for the programming language Python. All data were handled in Microsoft Excel (Microsoft) and analysed using RStudio 2021.09.1+372 (RStudio, 2021). All raw data used to generate the figures are shown in File S1.

## 3 RESULTS

**FIGURE 1:**
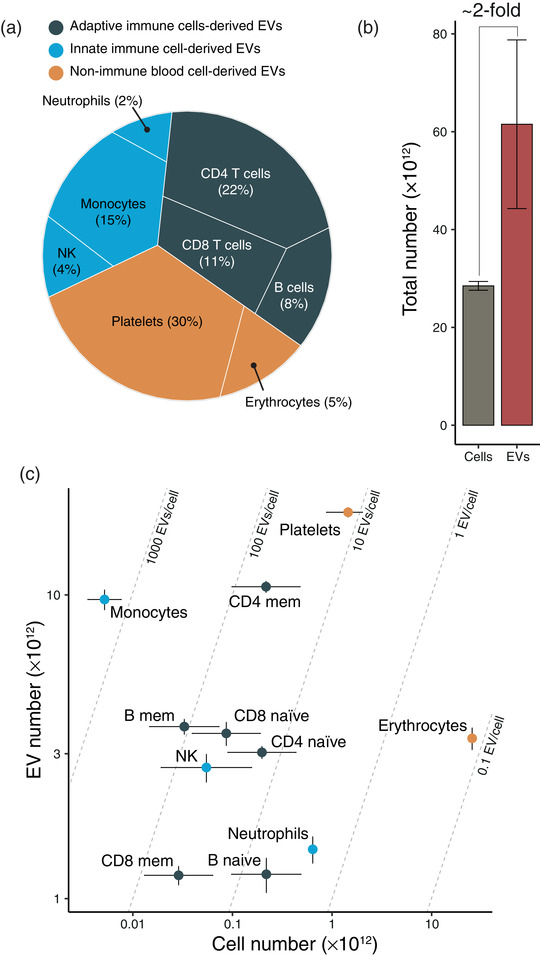
Estimation of cell‐specific plasma EV to cell ratio. **(a)** In healthy humans, plasma EVs are mainly derived from blood cells, and ∼41% is derived from the adaptive immune system cells (B, CD4, and CD8 cells), ∼23% from the innate immune system cells (monocytes, NK cells, and neutrophils), and ∼35% from non‐immune blood cells (erythrocytes and platelets). The remaining <1% is derived from cells in solid organs. A more resolved distribution map is shown in Figure S1a. The relative abundances are shown in parentheses. **(b)** The total number of parental blood cells and their plasma EVs. The overall plasma EV/cell ratio is ∼2. Error bar represents the estimate's standard deviation. **(c)** Plasma EV number versus cell abundance for human blood cell types. A graph with more resolved values for CD8 memory cells is shown in Figure S2a. Error bar represents the estimate's standard deviation.

The distribution of cell‐specific plasma EVs, based on plasma EV‐RNA data from 118 persons, indicate that adaptive immune system cells (B, CD4, and CD8), innate immune system cells (monocytes, NK cells, and neutrophils), and non‐immune blood cells (platelets and erythrocytes) derived EVs constitute, respectively, ∼41%, ∼23%, and ∼35% of the plasma EV pool (Figure 1a, a more highly resolved distribution map is shown in Figure S1). The remaining (<1%) plasma EVs are derived from solid organs (Li et al., 2020), and they were excluded from the downstream analyses. In a healthy person, historically defined as “male, 20–30 years of age, 70 kg and 170 cm” (Sender & Milo, 2021), the plasma EVs included in our analyses are derived from the 11 different blood cell types that constitute (29 ± 0.9) × 10^12^ cells. The adult human body (with the inclusion of platelets) consists of (31 ± 0.8) × 10^12^ cells – thus, ∼90% of the cells in the adult human body are included in the analyses.

Consistent with Johnsen et al. (Johnsen et al., 2019), we estimated the plasma EV concentration to (2.1 ± 0.4) × 10^13^EVs/L plasma, yielding a total number of (62 ± 17) ×  10^12^ plasma EVs and a ratio between plasma EVs and their parental blood cells of 2.2 ± 0.4 (Figure 1b). Figure 1c presents each cell type´s plasma EV and cell abundance. Dividing these two factors gives the cell‐specific EV‐to‐cell ratio, showing that high abundant cell types such as red blood cells and neutrophils were only represented by 0.1‐3 plasma EV per cell, while monocytes and lymphocytes were represented by 15–2000 EVs per cell (Figure 1c and Figure S2a). Thus, our analysis suggests that, at steady‐state, cell abundance is a poor predictor of EV abundance in that the cell‐specific EV‐to‐cell ratio spans four orders of magnitude.

We used the mean diameter of plasma EVs (112 ± 12 nm) and the EV mass density (Brennan et al., 2020; Colombo et al., 2014) to estimate the mass of a single plasma EV to 0.8 ± 0.3 fg. This agrees with previously reported values (Koliha et al., 2016; Son et al., 2015), suggesting that the combined mass of all plasma EVs in a human body is 51 ± 2 mg. The total mass of their parental blood cells is 2750 ± 140 g; thus, the plasma EVs are derived from a >50,000‐fold higher cell mass (Figure S2b). The mass of single cells varies considerably between cell types, for example, monocytes' average mass is 460 pg (SD range 418 – 506 pg) (Sender & Milo, 2021) and platelets' average mass is 8.6 ± 0.64 pg. The circulating mass of monocyte‐derived EV was estimated to be 0.3 ± 0.2% of the monocyte mass, and due to the relatively low platelet mass, their plasma EVs correspond to 0.1 ± 0.05% of the total platelet cell mass. On the other hand, erythrocytes are only represented by (1 ± 0.6) × 10^−4^% of their total cell mass (Figure S2c and d). Thus, our estimate suggests that the EV‐to‐cell mass ratio is <1% for all the cell types included in our analyses and spans three orders of magnitude.

**FIGURE 2:**
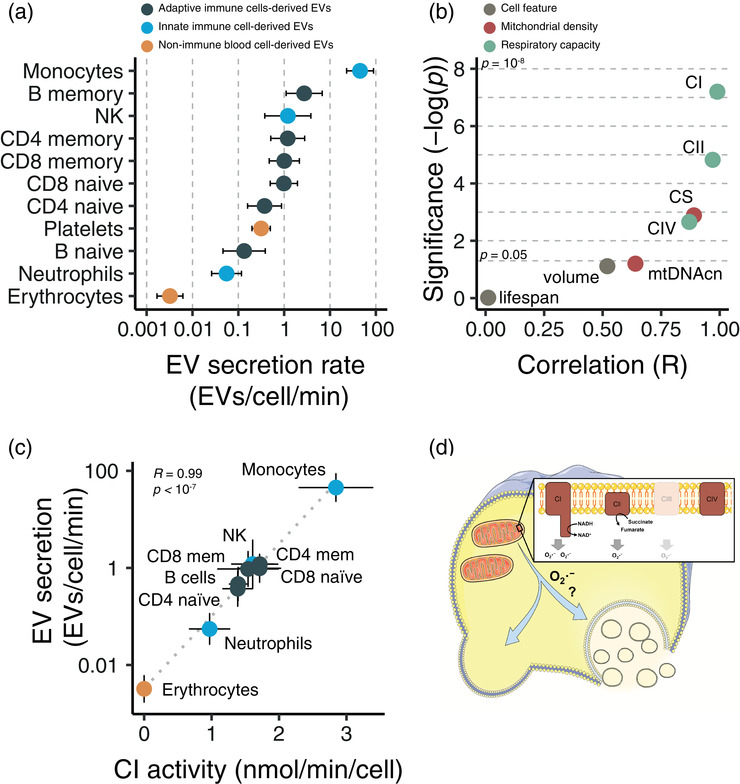
The estimated cell‐specific EV secretion rate correlates to mitochondrial metabolism. **(a)** The estimated cellular EV secretion rate for human blood cell types. A more detailed graph with values from the different CD8 memory cells is shown in Figure S2e. Error bar represents the estimate's standard deviation. **(b)** Statistical significance level versus Pearson's correlation of the estimated EV secretion rates and cell phenotype for human blood cell types. The dashed vertical lines indicate equal *p*‐values. The individual correlations are shown in Figure S3. **(c)** The logarithm of the estimated cellular EV secretion rate versus mitochondrial Complex I activities in human blood cell types. Error bar represents the estimate's standard deviation. The dashed line indicates the log‐linear correlation. **(d)** Schematic cell model of a proposed mechanism linking basal mitochondrial metabolism and EV secretion. CI, CII, CIII, and CIV indicate mitochondrial Complex I – IV, respectively.

At steady‐state conditions, EV secretion and elimination rates average out. Based on mouse data, as described in the method section, we estimated the EV mean plasma resident time (MRT) to 41 ± 7 min. This yields a plasma EV turnover rate to (1.5 ± 0.4) ×  10^12^ plasma EVs/min, corresponding to an EV secretion and elimination rate of 1.3 ± 0.5 mg /min. Assuming that the plasma EVs MRT is independent of the cell origin, we estimated the cell‐specific EV secretion rate for the individual blood cell types. Thus, erythrocytes had an estimated EV secretion rate of (3.2 ± 3.0) × 10^−3^ EVs/cell/min, while monocytes had an estimated EV secretion rate of 45 ± 21 EVs/min (Figure 2a and Figure S2e). To analyse whether the estimated EV secretion rates were associated with cell phenotypes, we made correlation analyses. We focused our analyses on cellular lifespan and volume, and as blood cells have a high range of mitochondrial densities, we also correlated with the cell‐type specific mitochondrial phenotype. While the logarithm of the estimated EV secretion rate did not correlate statistically significant with cellular lifespan (R = 0.01, *p =* 0.97, cell volume (R = 0.52, *p* = 0.08) or mitochondrial DNA copy number (R = 0.64, *p* = 0.06, Figure 2b and Figure S3a‐c), we observed a highly statistically significant correlation between the logarithm of the estimate EV secretion rate and activities of several mitochondrial enzymes reflecting mitochondrial density (citrate synthase (CS)) and respiratory capacity (Complex IV (CIV), CII and CI, Figure 2b). The estimated EV secretion rates were positively log‐linear correlated with CIV (Figure S3d, R = 0.87, *p* = 2.2 × 10^−3^), CS (Figure S3e, R = 0.89, *p* = 0.0013), CII (Figure S3f, R = 0.97, *p* = 1.5 × 10^−5^), and CI activities (Figure 2c, R = 0.99, *p* = 6.3 × 10^−8^). Thus, our cell‐specific analyses indicate that basal EV secretion rate is strongly correlated with mitochondrial metabolism.

## 4 DISCUSSION

Using reported values on plasma EV and cell abundances, we estimated plasma EV turnover for blood cells in healthy humans. Our results indicate that the blood cells' basal EV secretion rate correlates with their mitochondrial metabolism. Mitochondria are a major source of cellular reactive oxygen species (ROS), with mitochondrial Complex I having one of the highest ROS‐generating capacities (Starkov, 2008). There is ample evidence that increased cellular ROS levels increase EV secretion; for example, exercise (Wong et al., 2017) and hypobaric pressure at higher altitudes (Gaur et al., 2021) cause higher plasma EV concentrations (Brahmer et al., 2019; Frühbeis et al., 2015; Utermöhlen et al., 2019; Whitham et al., 2018) in healthy humans. Moreover, ultraviolet light generates ROS (de Jager et al., 2017) and increase EV secretion (Shen et al., 2020), and diseases, such as cancers, display high cellular ROS levels (Perillo et al., 2020), and cancer cell‐derived are circulating at high levels in patients (Minciacchi et al., 2015; Vitale et al., 2021). Furthermore, genetically modified mice with increased mitochondrial ROS production in adipocytes show elevated serum adipocyte‐derived EV levels (Crewe et al., 2021). Together, our analyses indicate that mitochondria‐produced ROS may be a driver of basal blood cell‐derived EV secretion in healthy humans (Figure 2d), and our results provide a foothold for a deeper understanding of EV biology in human health and disease.

Our estimated cell‐specific EV secretion rate spanned four orders of magnitude. The difference in secretion rates could be due to multiple factors, for example, cell‐specific EV biogenesis rates. Also, heterogeneity between subpopulations of a given cell type could influence the estimated EV‐secretions rates. Some of the high variance may also be explained by our analysis only covering RNA containing EVs; yet, cultured cells also show a high variation in EV secretion rate determined by surface marker analysis (Chiu et al., 2016; Son et al., 2015; Sung et al., 2020). Moreover, HeLa cells expressing a reporter for multivesicular body (MVB) fusion activity showed ∼5‐10 fusion events per min (Verweij et al., 2018), and with 10–15 intraluminal vesicles per MVB (Edgar et al., 2014; Villarroya‐Beltri et al., 2016), this yields an EV secretion rate of 50 ‐ 150 EVs/cell/min. Thus, data from several sources of cultured cells are consistent with our estimated EV secretion rates.

Our analysis suggests a total plasma EV secretion rate of 1.3 ± 0.5 mg/min in humans. This is only ∼100‐fold higher than the EV secretion rate estimated by pharmacokinetic modelling in mice (Matsumoto et al., 2019). Because a human's body mass is ∼2000‐fold higher than mouse and blood volume scales linearly with body mass in warm‐blooded animals (West et al., 1997), this indicates that the estimated plasma EV secretion rates do not scale with either mass or blood volume. However, the strong correlation between basal estimated cellular EV secretion rate and mitochondrial enzyme activities indicates that EV secretion rate may scale with metabolic rates. According to Kleiber's Law, the basal metabolic rate *B* of warm‐blooded animals scales to the ¾ power of body mass *M*, that is, *B ≈ M^3/4^* (Gillooly et al., 2001; Kleiber, 1947). Thus, compared to a 35 g mouse, the basal metabolic rate of a 70 kg human is ∼300‐fold higher. This is within an order of magnitude of the estimated plasma EV secretion rates. This is not meant to imply that basal metabolic rate accounts for all variations in plasma EV secretion rates, but it supports the notion that basal metabolic rates may be a significant contributor to plasma EV secretion.

Total energy expenditure is relatively stable in adult humans but declines in older adults (Pontzer et al., 2021). Similarly, the plasma EV level has been shown to decrease with age (Eitan et al., 2017). While the decline in metabolic rate may correlate with lower plasma EV levels, there is also a marked change in the distribution of immune and non‐immune blood cells with age (Rausser et al., 2021). CD8 T cell and platelet number, accounting for 39% of the total plasma EVs (Lai et al., 2021; Li et al., 2020, 2021), decrease significantly with age (Rausser et al., 2021). Thus, in addition to metabolic rate, the age‐related decline in plasma EV concentration may reflect the changing abundances of cell types with high EV secretion.

A limitation of our analyses is that they are based on computational deconvolution of RNA sequencing data of plasma EVs (Lai et al., 2021; Li et al., 2018, 2020, 2021). Similar approaches have been used to determine the cellular source of urine EVs (Zhu et al., 2021); however, the cell‐specific EV RNA loading might affect the analyses if, for example, there are cell‐specific EV RNA loading mechanisms (Garcia‐Martin et al., 2021). The estimates of plasma EV concentration, distribution of plasma EVs, and the cell‐specific mitochondrial phenotype are derived from separate human populations, and multiple factors such as ancestry, age, gender, and exposure to pathogens may confound our estimates. Moreover, the cell‐specific mitochondrial phenotypes were determined by measuring activities of separate mitochondrial enzymes. While the correlation of these individual measures to more integrated mitochondrial activity measurements is unknown, our finding that enzymes reflecting mitochondrial density and respiratory capacity are significantly correlated with estimated EV secretion rates indicate that mitochondria metabolism contributes EV secretion. In addition to this, our analyses did not include endothelial‐derived EVs. The estimated number of endothelial cells is ∼6 × 10^11^, which is similar to neutrophil abundance (Sender & Milo, 2021). Endothelial cells have a low mitochondrial volume fraction, and 85% of their ATP is generated by glycolysis (Bock et al., 2013). Consistent with the low mitochondrial metabolism, cultured endothelial EV secrete 0.07 EVs/cell/min (Garcia‐Martin et al., 2021), a number similar to our estimated EV secretion rate of neutrophils. Therefore, the endothelial‐derived plasma EV abundance is expected to account for ∼2% of the total pool of circulating EVs, which agrees with values obtained by flow cytometry (Augustine et al., 2014). Thus, although endothelial‐derived EVs are present in plasma and were omitted from our analyses, the estimated low endothelial‐derived plasma EV abundance indicates that this is not likely to affect the overall analysis significantly.

Whether the correlation between EV secretion rate and mitochondrial metabolism also holds for larger cell types, such as skeletal muscle cells, neurons, and adipocytes, remains to be determined. A complete description of EV turnover for other biological fluids may explain why, for example, the cerebrospinal fluid and interstitial fluid EV concentrations are 1/10^th^ (Martins et al., 2018) and 10‐fold (Miller et al., 2018) that of blood plasma. Nonetheless, our observation that mitochondrial metabolism is correlated to basal EV secretion rate in blood cells can inform many EV‐related questions such as how cell‐specific plasma EV levels should be interpreted and how cell content is sorted into EVs, forming the basis for a more profound understanding of EV biology in human health and disease.

## AUTHOR CONTRIBUTIONS

Martin Auber: Formal analysis; Investigation; Methodology; Visualization; Writing – review & editing. Per Svenningsen: Conceptualization; Data curation; Formal analysis; Funding acquisition; Investigation; Methodology; Project administration; Visualization; Writing – original draft; Writing – review & editing

## CONFLICT OF INTEREST

None.

## Supporting information

SUPPORTING INFORMATION

SUPPORTING INFORMATION
