# Supplementary material for: An estimate of extracellular vesicle secretion rates of human blood cells
Source: J Extracell Biol. 2022 Jun 2;1(6):e46. doi: 10.1002/jex2.46 (PMC11080926; doi:10.1002/jex2.46)
Supplement: Supplementary file 1 — SUPPORTING INFORMATION [file JEX2-1-e46-s002.pdf]

Supplement Figure 1

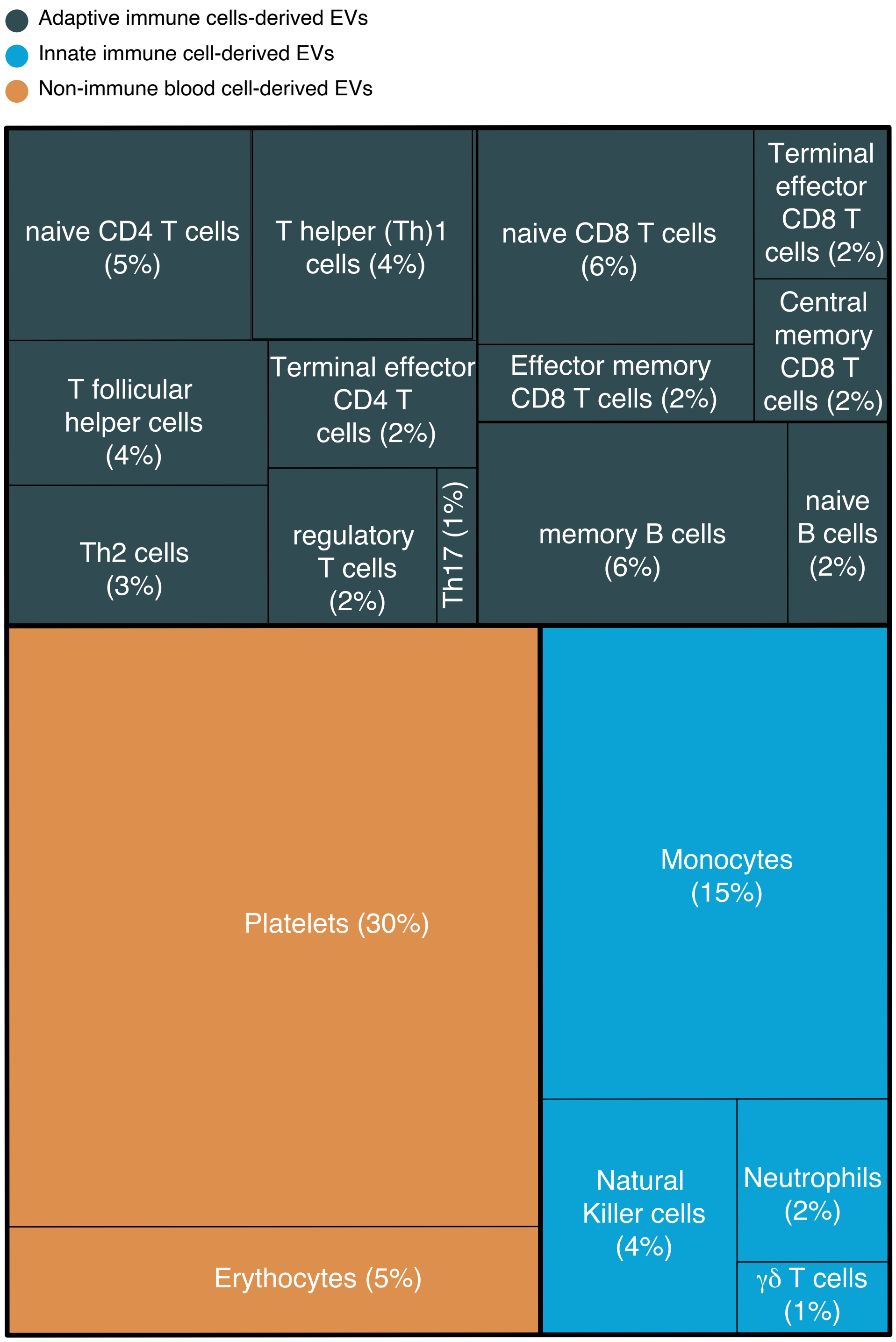

Treemap of the distribution of human plasma EVs based on RNA sequencing data.
The raw data are available in Supplementary File 1.
The relative abundances are shown in parentheses.

Supplement Figure 2

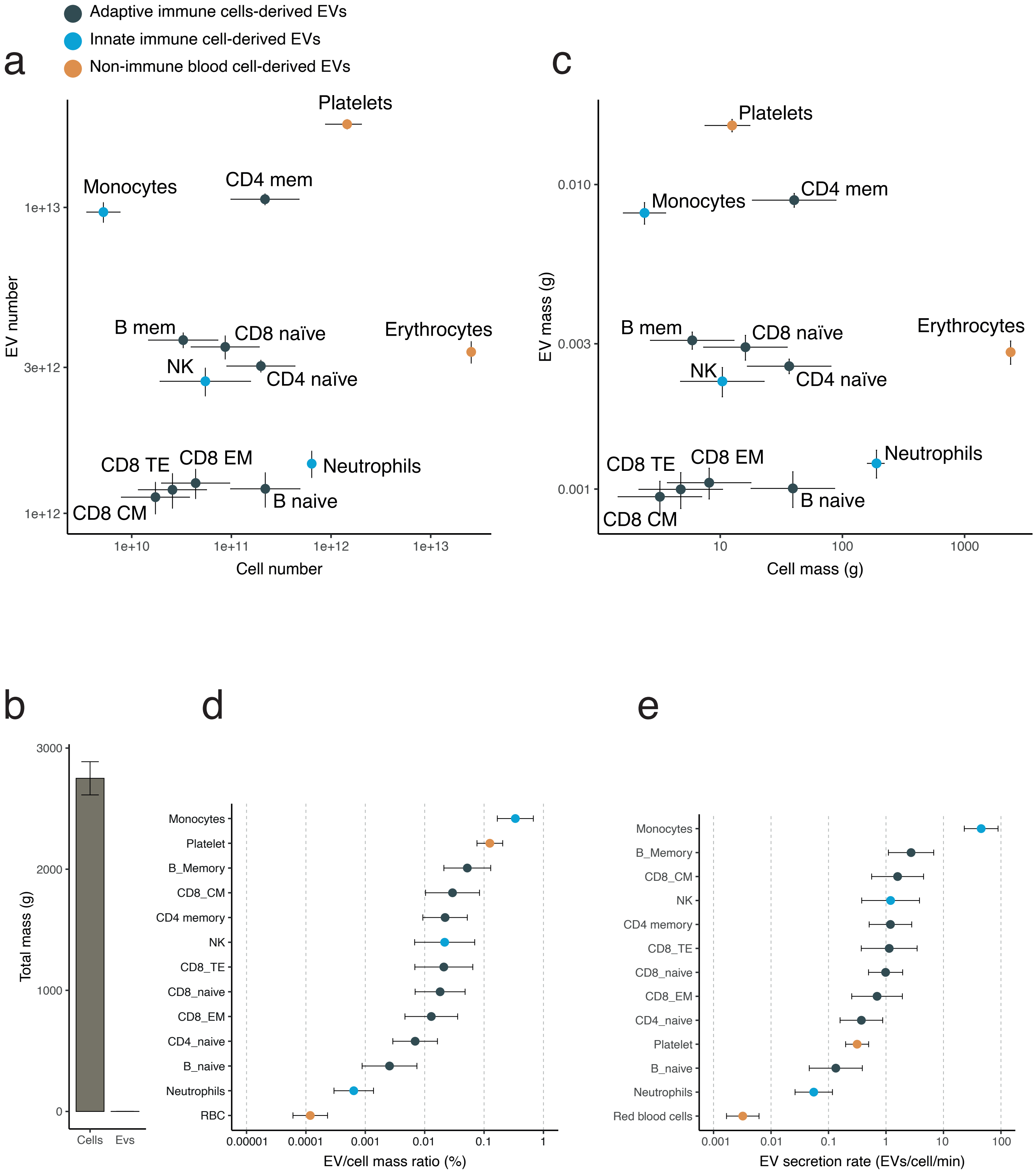

(a) Extended version of the graph shown in Figure 1c.
(b) The estimated total mass of plasma EVs and their parental cells.
The overall EV/cell mass ratio > 50,000.
(c) The cell-specific plasma EV mass versus the cellular mass for human blood cell types.
(d) The individual cell type's plasma EV/cell mass ratio is shown in percent.
(e) An extended version of Figure 2a presenting the individual cell types' plasma EV secretion rates.

Supplement Figure 3

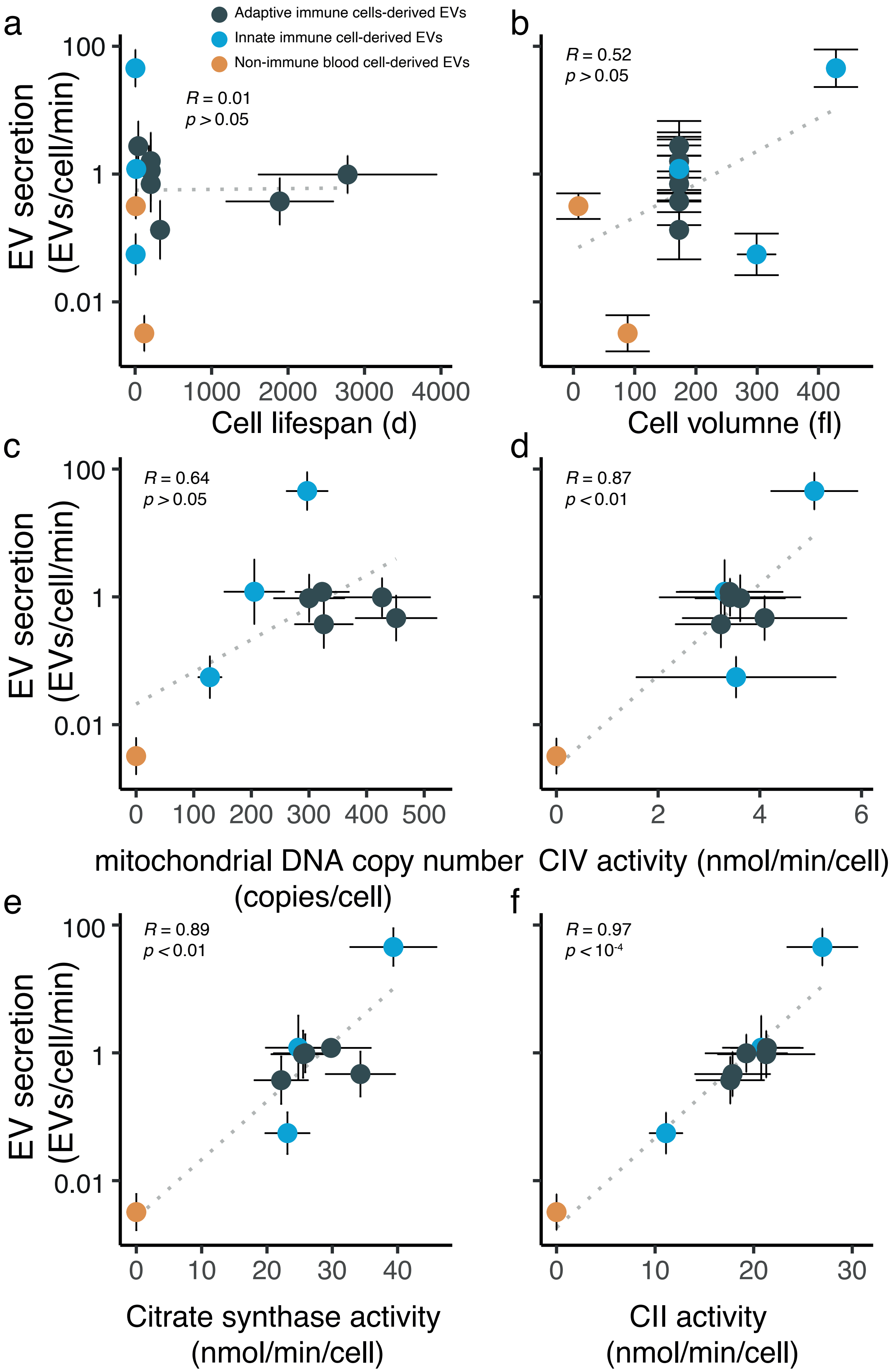

Correlation between the logarithm of the estimated cellular EV secretion rates and (a) cell lifespan, (b) cell volume, (c) mitochondrial DNA copy number, (d) mitochondrial Complex IV activity, (e) mitochondrial citrate synthase activity, and (f) mitochondrial Complex II activity for human blood cell types.
